# The application of a neural network to predict hypotension and vasopressor requirements non-invasively in obstetric patients having spinal anesthesia for elective cesarean section (C/S)

**DOI:** 10.1186/s12871-020-01015-9

**Published:** 2020-05-01

**Authors:** Irwin Gratz, Martin Baruch, Magdy Takla, Julia Seaman, Isabel Allen, Brian McEniry, Edward Deal

**Affiliations:** 1 Cooper University Hospital, 1 Cooper Plaza, Camden, NJ 08103 USA; 2 Caretaker Medical, Charlottesville, VA USA; 3 Quahog Research Group, Oakland, CA USA; 4 University of California – San Francisco, San Francisco, CA USA

**Keywords:** Arterial stiffness, Cesarean section, Finger cuff, Hypotension, Neural network, Non-invasive, Predictive algorithm

## Abstract

**Background:**

Neural networks are increasingly used to assess physiological processes or pathologies, as well as to predict the increased likelihood of an impending medical crisis, such as hypotension.

**Method:**

We compared the capabilities of a single hidden layer neural network of 12 nodes to those of a discrete-feature discrimination approach with the goal being to predict the likelihood of a given patient developing significant hypotension under spinal anesthesia when undergoing a Cesarean section (C/S). Physiological input information was derived from a non-invasive blood pressure device (Caretaker [CT]) that utilizes a finger cuff to measure blood pressure and other hemodynamic parameters via pulse contour analysis. Receiver-operator-curve/area-under-curve analyses were used to compare performance.

**Results:**

The results presented here suggest that a neural network approach (Area Under Curve [AUC] = 0.89 [*p* < 0.001]), at least at the implementation level of a clinically relevant prediction algorithm, may be superior to a discrete feature quantification approach (AUC = 0.87 [*p* < 0.001]), providing implicit access to a plurality of features and combinations thereof. In addition, the expansion of the approach to include the submission of other physiological data signals, such as heart rate variability, to the network can be readily envisioned.

**Conclusion:**

This pilot study has demonstrated that increased coherence in Arterial Stiffness (AS) variability obtained from the pulse wave analysis of a continuous non-invasive blood pressure device appears to be an effective predictor of hypotension after spinal anesthesia in the obstetrics population undergoing C/S.

This allowed us to predict specific dosing thresholds of phenylephrine required to maintain systolic blood pressure above 90 mmHg.

## Background

Intraoperative hypotension has been reported to occur in between 5 to 99% of cases depending on the specific surgical population and it is associated with complications that can harm patients [1]. A particularly vulnerable population are obstetric patients undergoing spinal anesthesia for Cesarean sections (C/S).

Spinal anesthesia in this population is associated with hypotension incidence as high as 70% and hypotension remains a common and clinically-important problem that is associated with morbidity for both mother and child. Even brief episodes of hypotension can result in lower fetal Apgar scores and acidosis [2–4].

Detection and management of maternal hemodynamic instability during C/S remains a primary clinical and research focus as early recognition can enhance clinical decision making [5]. The latest developments in monitoring of arterial waveforms invasively allow for the prediction of hypotension to possibly improve patient outcomes [6, 7]. However, prediction of hypotension based on a noninvasive technique would expand our monitoring and diagnostic capabilities. For the purpose of the study, hypotension in this population was defined as a systolic blood pressure < 90 mmHg, this blood pressure was chosen because it is our institution’s current standard for the C/S population. Furthermore, it aligns with other investigators, the consideration being that spinal sympathectomy primarily affects systolic blood pressure [8]. Additionally, systolic and diastolic blood pressures are the two most often reported parameters used in both clinical practice and clinical research studies as they are proven markers of cardiovascular disease [9].

We evaluated the CareTaker® (CT) continuous noninvasive blood pressure device (Caretaker Medical LLC, Charlottesville, Virginia) which has been described in detail elsewhere [10, 11]. Briefly, the CT is a physiological sensing system that communicates physiological data wirelessly via Bluetooth. The device uses a low pressure (35–45 mmHg), pump-inflated, cuff surrounding the middle phalange of the middle finger that pneumatically couples arterial pulsations via a pressure line to a custom-designed piezo-electric pressure sensor for detection and analysis.

The use of pulse analysis of the arterial pressure pulse offers a potential tool to investigate physiological markers for the prediction of hypotension, without impacting clinical workflow. A plausible physiological candidate for predicting the likelihood of hypotension in C/S patients undergoing spinal anesthesia is arterial stiffness (AS), which has been investigated in the separate contexts of hypotension and pregnancy, and can be assessed using pulse contour analysis [12]. Recent work on the prediction of imminent hypotension has focused on identifying changes in the variability of physiological signals, among them AS [13, 14]. Variability changes are due to compensatory mechanisms in the cardiovascular system as it attempts to maintain stability [10, 15]. In the context of pregnancy, significant longitudinal changes in AS have been documented [13]. It is therefore reasonable to investigate whether underlying compromised physiological compensatory reserve can be predicted prior to spinal anesthesia induction.

The CT is FDA-cleared for the measurement of heart rate, continuous noninvasive blood pressure, and respiration. Blood pressure monitoring is accomplished via a pulse contour analysis algorithm called Pulse Decomposition Analysis (PDA), which analyzes the component pulses, specifically the left ventricular ejection pulse (P1) and its reflections, the renal reflection pulse (P2) and the iliac reflection pulse (P3), that constitute the arterial pressure pulse [16]. Part of the PDA framework is the AS parameter which quantifies the spectral content of the arterial pressure pulse that is due to the component pulses [10]. The spectral content in turn is related to arterial stiffness as it is the mechanical filtering of the arterial wall that determines to what extent the structure of the component pulses is resolved. As determined in other studies, this filtering limits the upper observable frequency components in the peripheral arterial pressure pulse to approximately 20 Hz [17]. Preliminary validation tests indicate that the AS parameter tracks expected trends after the introduction of vaso-active agents as well as age-related population trends [10].

The aim of the present study was to develop a preoperative model that could predict the development of severe post spinal hypotension noninvasively using AS as a hemodynamic marker.

## Methods

This study was approved by the Institutional Review Board of Cooper University Hospital (IRB #17–119) and all patients provided written informed consent. The study population was a subset of a larger study which compared the agreement of blood pressures obtained from the CT non-invasive blood pressure measurement device to those from intermittent oscillometric cuff inflations during abdominal and obstetric surgeries.

Forty nine patients (> 34 weeks gestation) with an American Society of Anesthesiologists status II who were undergoing elective C/S under spinal anesthesia were enrolled in this study. All patients had an intravenous catheter started in the preoperative preparation room. Lactated Ringer’s solution was slowly infused to keep the vein open.

Measurements were started in the preoperative preparation room approximately 90 min prior to initiation of spinal anesthesia and continued throughout the entire procedure. The CT device provides physiological data, including systole, diastole, mean arterial pressure (MAP), heart rate and the AS parameter, on a beat-by beat basis. The data is transmitted wirelessly from the central, wrist-worn, processing unit to a nearby Android-based tablet (Samsung Galaxy Tab. A, Samsung Group, Seoul, South Korea) that is part of the FDA-cleared CT system.

### Anesthesia procedure

For the purposes of this study hypotension is defined as systolic blood pressure < 90 mmHg. In this prediction study, a 30-min data window after the start of data collection was used for analysis, starting approximately 90 min prior to induction. All patients underwent spinal anesthesia with a 24-gauge spinal needle in sitting position based on the classic midline method with the administration of 10.5 mg bupivacaine solution 0.5%. We quickly (within 3 min) placed patients in the supine position after the injection and this practice should have limited the development of hypotension immediately after the spinal injection. Intra-operatively, systolic blood pressure was maintained above 90 mmHg with boluses of phenylephrine (100 mcg). Boluses were repeated at 5 min intervals until a systolic blood pressure of > 90 mmHg was achieved as per our standard protocol. The blood pressure and heart rate were measured, separate from the beat-by-beat CT-based measurements, using an upper arm cuff (Critikon Soft-Cuf, model SFT-A2-2A, GE Healthcare, Chicago, Illinois, USA) initially every 2 min for 10 min, and every 5 min thereafter. All patients were pre-hydrated with 1000 ml of lactated Ringers just prior to the spinal injection. The anesthesia level was determined in all patients and was between thoracic levels 4 to 6 as measured by a pin prick. All patients were placed in the left lateral position to ensure avoidance of compression of the vena cava by the gravid uterus.

### Arterial stiffness assessment

The AS parameter that is part of the pulse analysis PDA framework has been described in detail elsewhere [10]. Briefly, the parameter quantifies the spectral content of the arterial pressure pulse envelope and is driven primarily by the resolution of the section of overlap of the renal pulse (P2), and the iliac pulse (P3). This section incorporates the pulse region that was examined by others and was found to correlate with expected age- and drug-related changes in arterial stiffness [18].

Visual examination of AS data of patients scheduled to undergo spinal anesthesia as part of a C/S procedure suggested that subjects who later required higher phenylephrine dosages to stabilize their persistent hypotension exhibited larger variability in the 30-min time-window 90 min prior to induction. Specifically, these patients would exhibit AS modulations, sometimes distinctly oscillatory, with time scales on the order of 3 min. From these observations arose the hypothesis that the likelihood of post-induction severe hypotension, defined by the need for the administration of significant dosages of phenylephrine, albeit at an as yet underdetermined threshold, could be predicted by a measure of the amplitude or duration of the observed modulations in the AS data. The benefit of choosing this indication of hypotension, as opposed to for example the time duration for which systole < 90 mmHg, is that, even on cursory examination, it provided less ambiguity than the interpretation of blood pressure readings near a threshold, which clinicians based on their extensive experience do routinely, would have introduced.

### Signal pre-processing

**Fig. 1:**
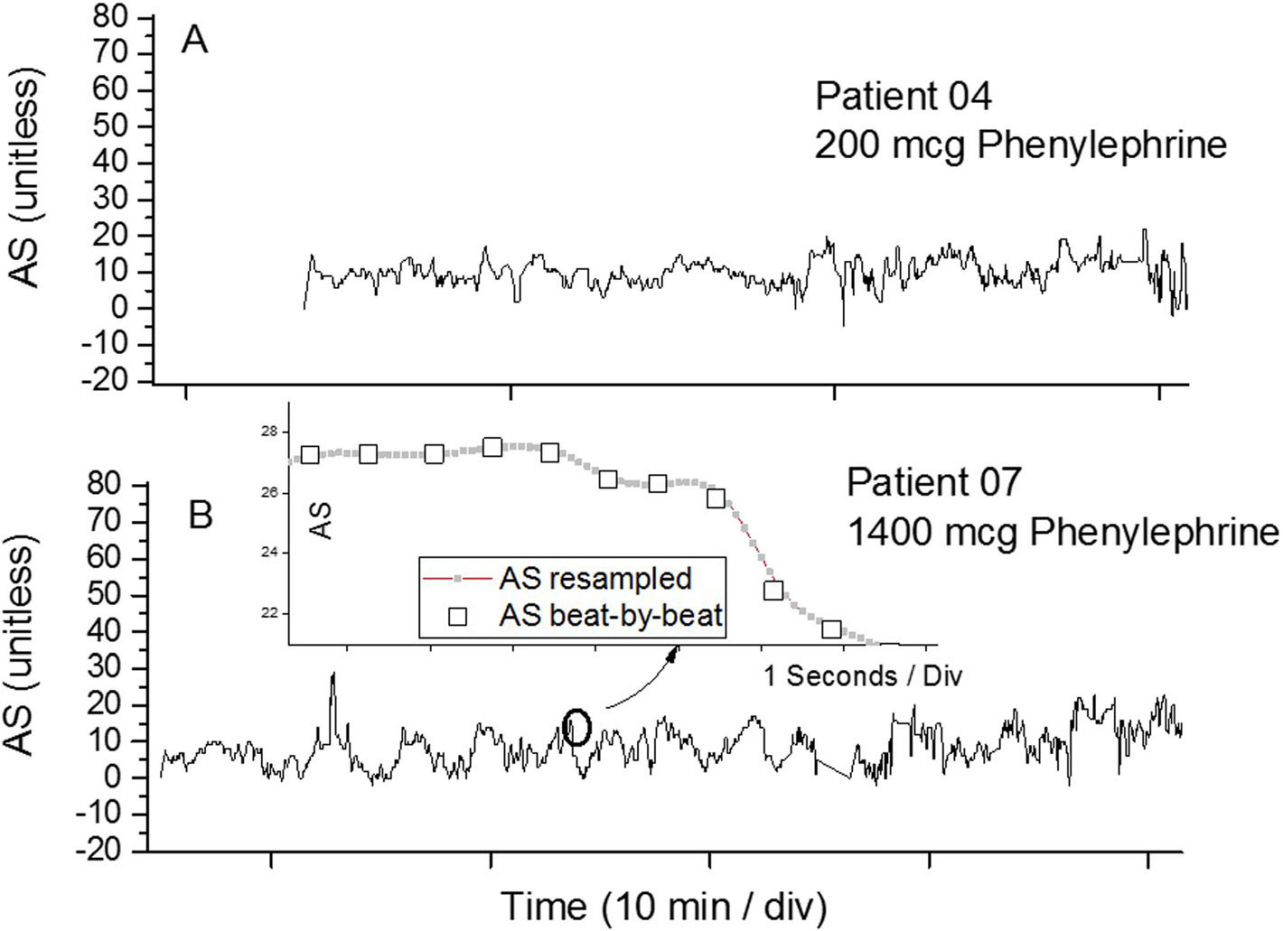
**a**. Time evolution of the AS response of patient 04, 60 min prior to induction, who subsequently required minimal phenylephrine intervention, 200 ml. **b**. Time evolution of the AS response of patient 07, 60 min prior to induction, who subsequently required significant phenylephrine intervention, 1400 ml. The graph of patient 07 also shows an inset with an expansion and overlay of about 15 s of the original beat-by-beat AS data as well as the AS data re-sampled at 10 Hz

As was stated above, the AS data is provided by the CT system on a beat-by-beat basis, i.e. at a non-uniform heart rate. For most signal-processing approaches, non-uniformly spaced data presents a significant challenge. This includes correlation schemes which involve the digital mapping of data sections onto other data sections, with highly unpredictable results if data time intervals are not equal. The data were therefore linearized at a frequency to preserve the spectral content of the inter-beat variations, which in this cohort, at heart rates between 70 and 103 bpm, resides in the 1–2 Hz frequency band. To assure oversampling at a factor of 5, as opposed to the Nyquist limit of 2, times the highest frequency embedded, a linearization frequency of 10 Hz was chosen and implemented using spline resampling. Figure 1b, which displays an example of the time evolution of the AS response of patient 07, also displays a highly enlarged, about 9 s, data section for comparison of the original and the resampled data trace, which without the enlargement would be indistinguishable.

Since inspection of the observed modulations revealed limited coherency and because of the very low frequency regime involved, spectral analysis approaches were not considered. Instead the data were analyzed using autocorrelation spectra that provide information on signal coherence. Cross- and auto-correlation-based analyses have been used for pattern recognition in the context of physiological signals such as, for example, blood pressure, heart rate variability, and respiration [19–21]. Specifically, for a signal that is self-coherent on some time scale, the autocorrelation will exhibit correlation coefficients of significant amplitude and the coefficients will change sign, i.e. exhibit zero-crossings, with different lag-times as the signal’s time components constructively and destructively interfere with each other.

For each patient, the autocorrelation spectrum was calculated for a 2000 s window, or about 33 min of 10 Hz resampled AS data, corresponding to 20,000 data points. This yields a same-sized autocorrelation spectrum with time lags from 0 to 2000 s at the resampled resolution. Visual inspection was used to select the specific analysis window for each patient so as to avoid data in the patient session that was clearly motion artifact-contaminated due to the patient settling in, which usually occurred in the first 5 min of recorded data.

### Single-feature extraction and classification assessment

The observed modulations were quantified by integrating over the absolute-value of the lag times of the autocorrelation spectra from 100 s to the maximum time lag of 2000 s, the goal being to isolate the observed longer-time scale modulations from shorter-range lag times. Analyses were performed in Matlab 2017b (MathWorks, Natick, MA, USA).

Using the value obtained from the absolute AS autocorrelation area integration for each patient as a metric, ROC analyses were used to assess whether the metric could classify those patients that developed severe hypotension, characterized by requiring higher dosages of phenylephrine, from those who developed no or less severe hypotension, as indicated by lower dosages or the absence of any drug administration. The cumulative phenylephrine dosage administered to the respective patient was considered, irrespective of stepped administrations. Classification accuracy was assessed for different phenylephrine dosage thresholds.

### Neural network classification assessment

In a separate analysis approach, a single hidden layer neural network (NN) of 12 nodes, using back-propagation and gradient descent, was tasked with the same classification for the different phenylephrine thresholds. The basic configuration of the fully interconnected feed-forward NN used here and the basic equations describing its functionality are as follows:

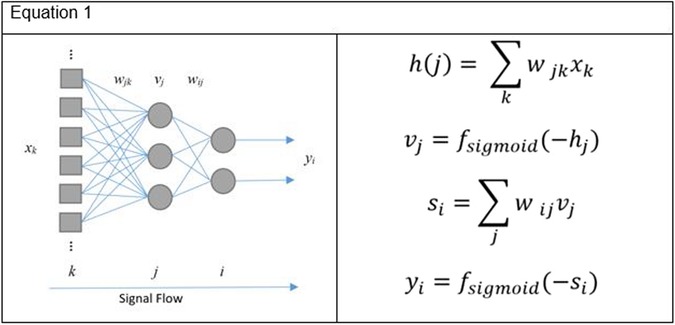



The input data elements x_k_ are individually weighted, summed, and the summation is the input to a sigmoidal activation function before the output is submitted to hidden nodes v_j_, whose outputs in turn are weighted, summed and presented to another activation function before submission to the output nodes y_i_. Additional hidden layers, with the commensurate interconnections, weighting and summing activating of each layer’s outputs etc., can be inserted to analyze performance as a function of input signal combinations.

The motivation here was to assess the classification capability of a “black box” approach that would have access, in contrast to the single-metric approach described above, to any number and combination of distinguishing features hidden in the data. If a significant number of such features were to exist, the classification performance of the NN would be expected to significantly exceed that of the single-metric approach. Classification performance was assessed using distributions and means of classification runs, the number of which was determined based on error and classification accuracy convergence. The choice of nodes, as well as the single-layer configuration, was arrived at by an analysis of the error as well as the classification accuracy of differently sized networks at different phenylephrine thresholds in order to address the potential of over-fitting.

The sample size required to estimate an area under the curve (AUC) of 0.85 ± 0.025 was calculated to be at least 33 patients, assuming a Type 1 error of 0.01, a power of 0.95, and the same number of mild and severe hypotension cases, i.e. an allocation ratio of 1 [22].

### Prediction attempt based on comorbidities and pre-Op systole

The possibility of predicting severe hypotension based on baseline patient conditions, comorbidities or pre-op systolic blood pressure cuff measurements was investigated.

## Results

Forty-nine patients were monitored as part of the study, with data from 45 patients included in the analysis. Table 1 lists patient population characteristics as well distributions of patient conditions and comorbidities. The following four patients were excluded from the original 49: One patient did not receive a spinal injection, the pre-injection data session from one patient was too short and two other data sessions were too compromised due to motion artifacts. For the 45 patients considered, which includes 4 patients who did not require the administration of phenylephrine, the mean dosage was 462 mcg, standard deviation (SD) 299 mcg, to treat hypotension post induction. No other vasopressor was used.

**Table 1:** Patient characteristics

Age (years)	
Mean (SD)	31.7 (4.72)
Range	21–44
Height (cm)	
Mean (SD)	160.9 (6.83)
Range	149.9–180.3
Weight (kg)	
Mean (SD)	98.7 (20.48)
Range	62.6–136.5
BMI (kg/m^2^)	
Mean (SD)	37.8 (7.76)
Range	23.7–55.2
Pre-op systole (mmHg)	
Mean (SD)	128.9 (19.14)
Range	74–194
Systole at induction (mmHg)	
Mean (SD)	131.2 (18.18)
Range	96–202
Systole at + 10 min (mmHg)	
Mean (SD)	115.4 (21.18)
Range	66–215
Minimum systole (mmHg)	
Mean (SD)	98.13 (14.89)
Range	61–137
Minutes until minimum systole	
Mean (SD)	12.68 (8.48)
Range	2–39
Medical history	
Prior C/S (Y/N), n	9/36
Hypertension (Y/N), n	6/39
Diabetes mellitus (Y/N), n	6/39
Chronic obstructive pulmonary disease (Y/N), n	0/39
Atrial fibrillation (Y/N), n	0/39

Figure 1a displays an example of the time evolution of the AS response, linearized and resampled to a rate of 10 Hz, of Patient 04, 90 min prior to induction, who subsequently required minimal phenylephrine intervention, 200 mcg, while Fig. 1b displays comparable results for Patient 07 who required 1400 mcg to stabilize her hypotension.

**Fig. 2:**
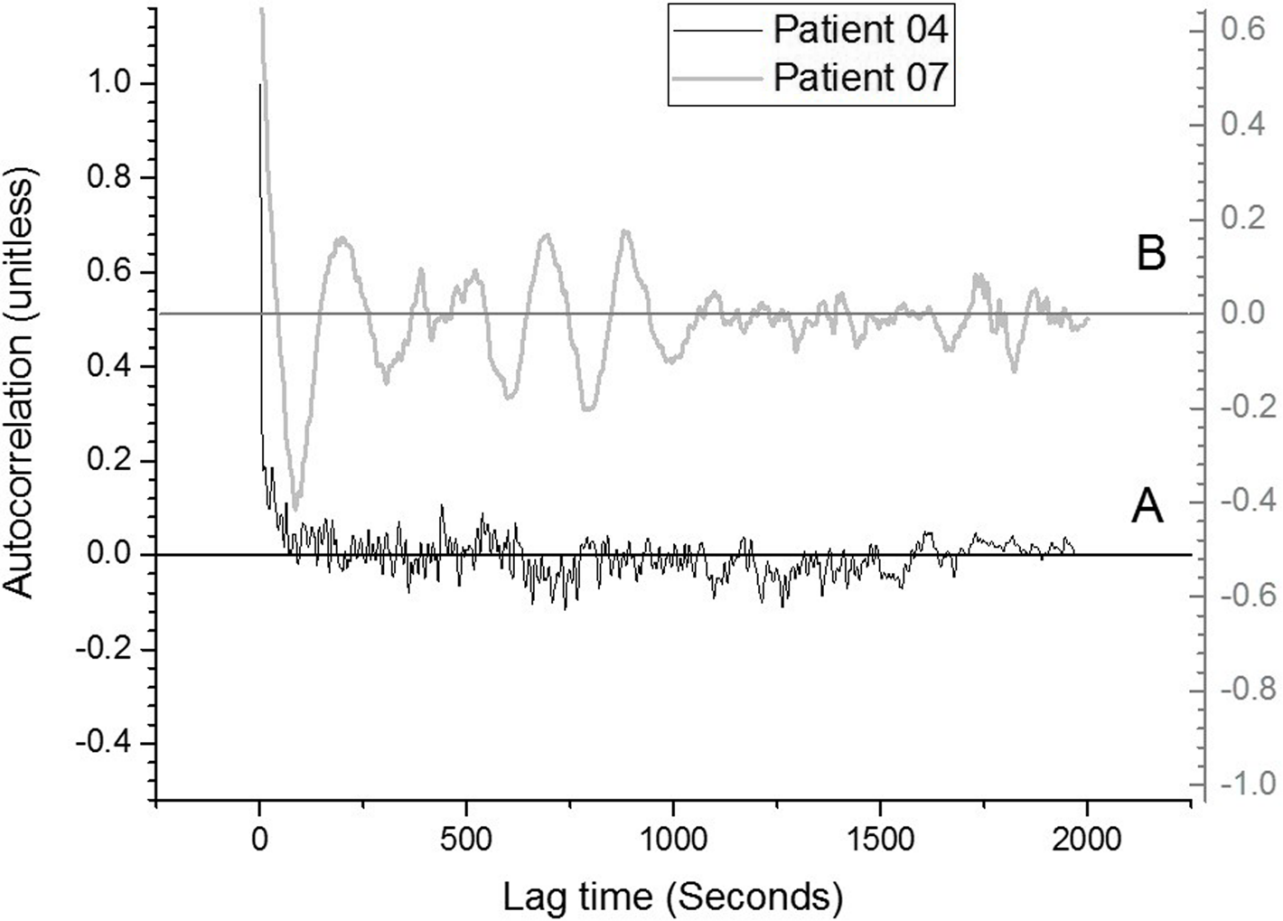
Black trace (**a**): Normalized AS autocorrelation spectrum of patient 04 (Fig. 1a, 200 mcg) suggests minimal coherence in the AS signal due to highly unequal and low-amplitude positive and negative correlations. Gray trace (**b**): Normalized AS autocorrelation spectrum of patient 07 (Fig. 1b, 1400 mcg) suggests high coherence in the AS signal. Spectra are offset from each other for clarity

Examination of the AS autocorrelation spectrum of Patient 04, Fig. 2 (trace A), suggests minimal coherence in the AS signal as the excursions of the coherence amplitude from zero are very small. The autocorrelation spectrum for Patient 07, Fig. 2 (trace B, offset from trace A for clarity), displays a more coherent response. Here the positive and negative correlation coefficients display oscillatory and significant amplitudes, suggesting that significant coherent signal components are present with distinct phase relationships.

**Fig. 3:**
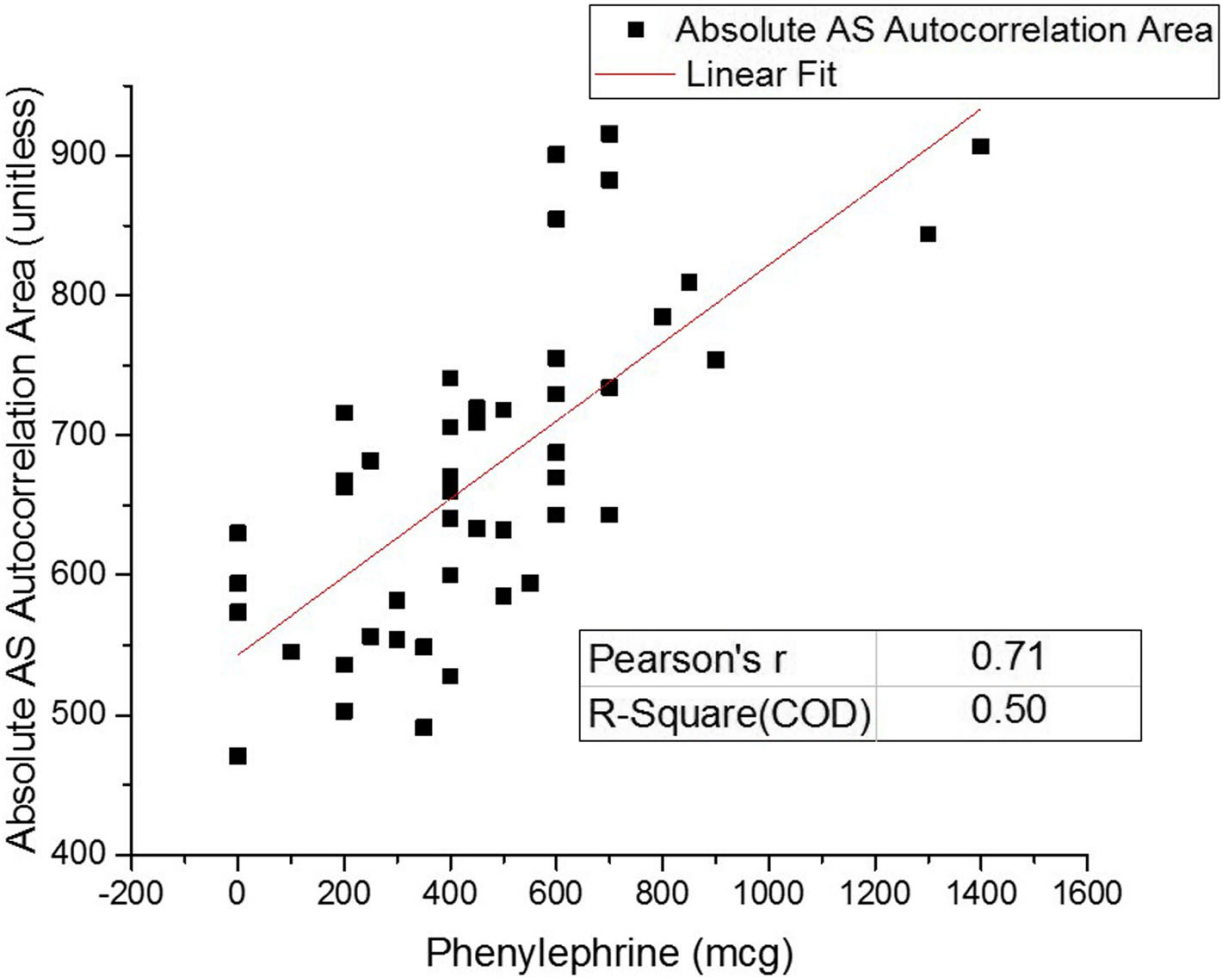
Graph of the result of the absolute autocorrelation value integration, for each patient, as a function of the total phenylephrine dosage administered to the respective patient

In order to parameterize the observed coherence response, an integration over the absolute value of each patient’s coherence spectrum, starting from a time lag of 100 s, was performed, as previously described. Figure 3 displays the result of performing the absolute autocorrelation value integration, for each patient, and graphing the results as a function of the total phenylephrine dosage administered to the respective patient.

**Fig. 4:**
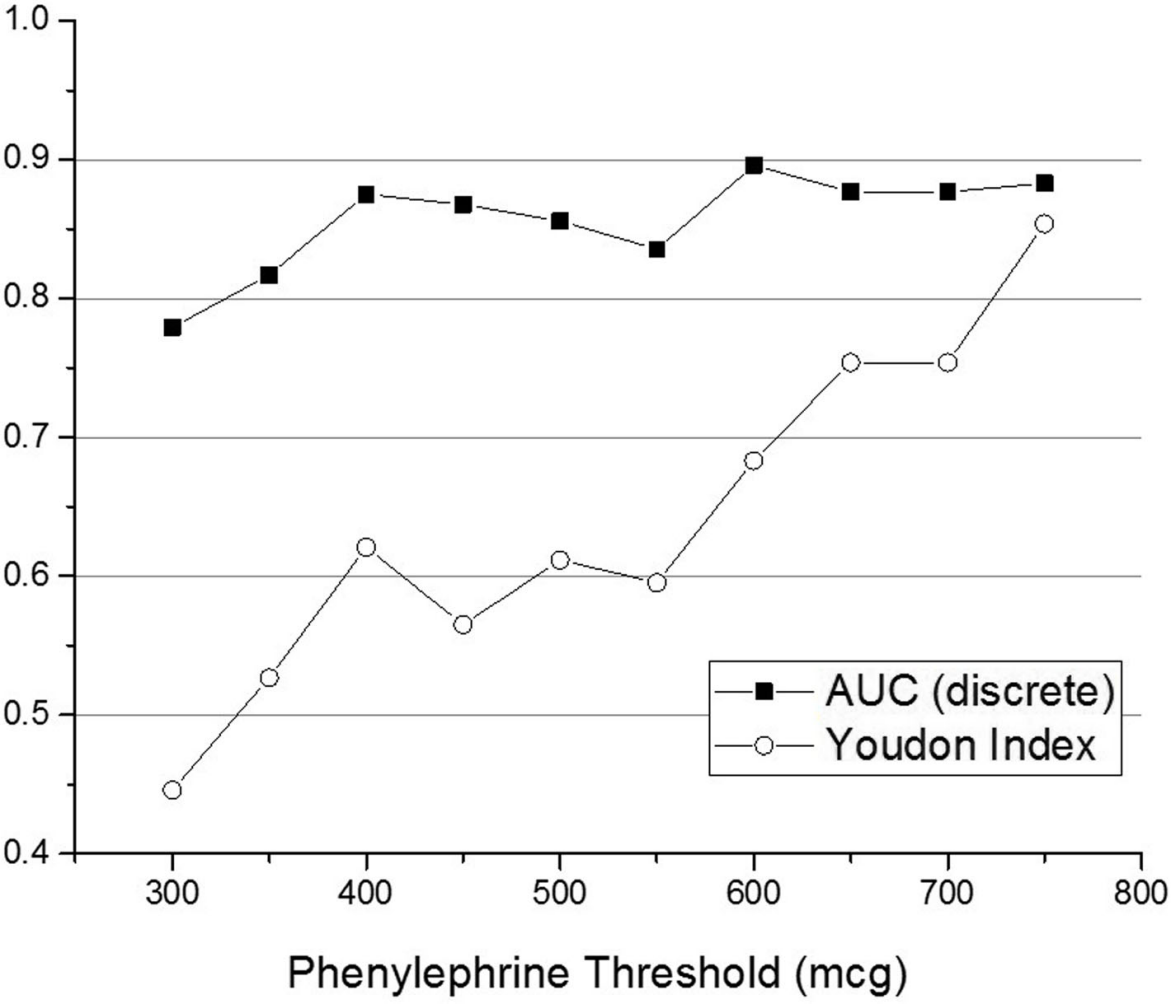
Youden index (open circles) and AUC values (solid squares) as a function of phenylephrine dosage threshold for the single-feature analysis using the absolute autocorrelation value integration as a metric

**Fig. 5:**
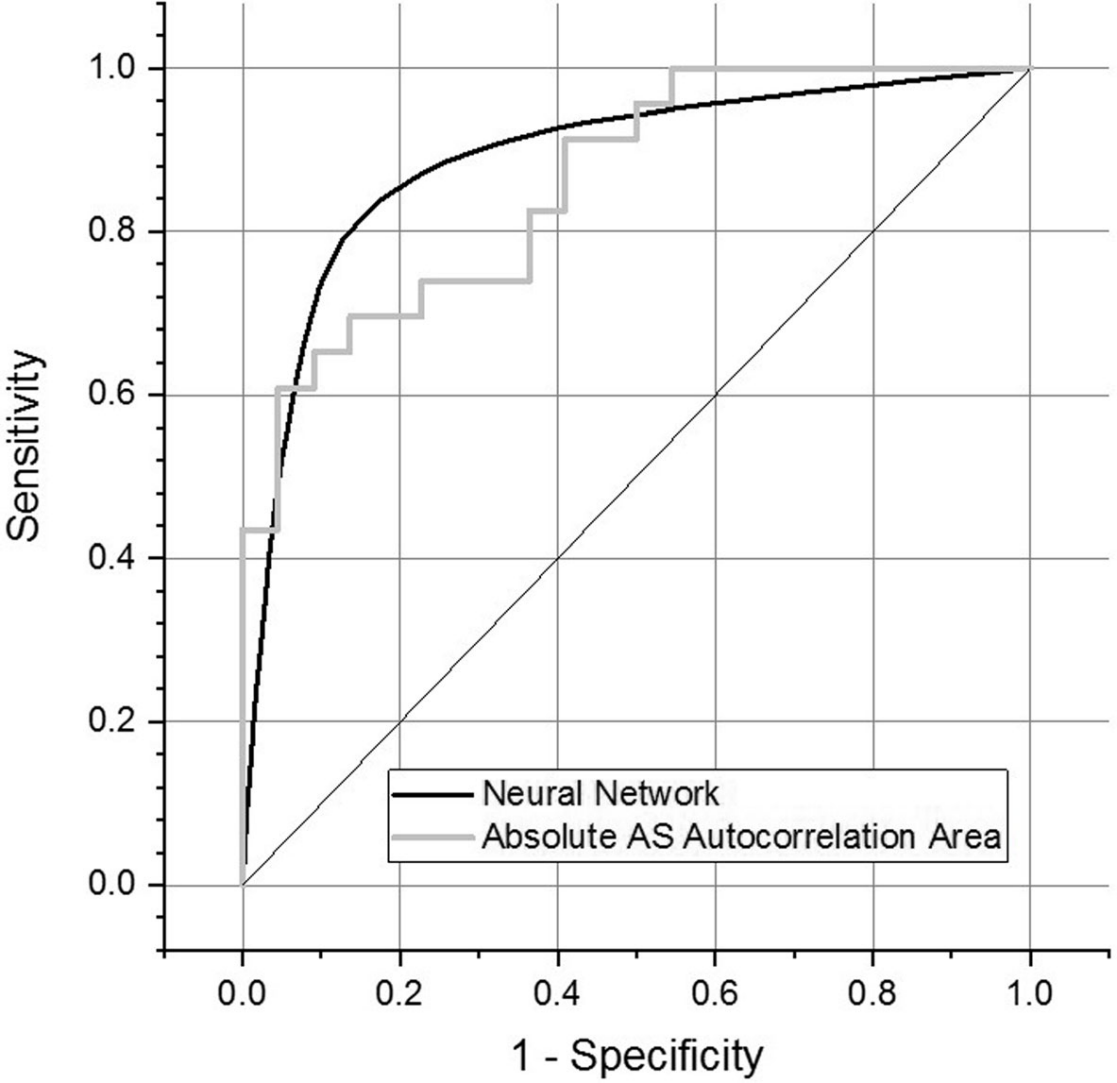
Light gray trace: ROC analysis based on phenylephrine dosage <=400 mcg or > 400 mcg. AUC = 0.87 for autocorrelation area. Solid black line: ROC analysis based on average of 500 runs of 12 node NN based on phenylephrine dosage <=450 mcg or > 450 mcg, AUC = 0.89

A ROC analysis was performed to assess the ability of the absolute AS autocorrelation area metric, obtained 90 min pre-induction, to predict the likelihood of a given patient developing severe hypotension post-induction. In order to establish the optimal discrimination threshold, the Youden index, which is defined as sensitivity + specificity − 1 and provides a summary measure of a discriminatory test, was used. The AUC and resulting sensitivity/specificity were determined for different dosage thresholds of the data and are presented in Fig. 4, which presents the results for the Youden index (open circles) and AUC (solid squares) as a function of phenylephrine dosage threshold. The local maxima in the Youden index and AUC analysis suggest that 400 mcg is an optimal ROC threshold. The resulting ROC, with an AUC = 0.87 (*p* < 0.001), is presented in Fig. 5 (light gray curve). Specificity and sensitivity were calculated from the Youden index corresponding to that threshold and, respectively, are 0.68 and 0.93.

In order to validate these results and obtain a more comprehensive assessment of the classification potential of the feature set characterizing the AS autocorrelation spectra with regard to predicting severe hypotension, the spectra were submitted for classification to a 12-node single hidden layer NN. A detailed analysis, described below, was performed to assess the optimum number of nodes as well as the number of layers.

The following hyper parameters were used: the batch sized equaled the sample size, i.e. Fourty-five patient data sets; a learning rate of 0.01 was chosen since speed optimization was not a concern; a log-sigmoid transfer function was used for node activation. An analysis was performed to address the potential of over-fitting by assessing the classification accuracy and the classification error as a function of the number of network nodes and network layers.

Since training of a given NN amounted to gradient searches in very high dimensional spaces, some searches would terminate in local minima, with commensurately poor classification as reflected in low AUC values and significant classification errors. Other training runs would avoid local minima and yield good, in rare cases perfect, classification. Five hundred training runs were performed for each dosage threshold with randomized initialization of network weights. For each run, training data sets, validation data sets and test data sets were randomly chosen based on the ratios, respectively, of 0.7, 0.15, and 0.15. The optimum number of training runs was determined by extending their number until the standard deviation in the errors of a series of runs was approximately 1/10 the maximum range of errors observed at a fixed phenylephrine dosage. Each run was terminated once the validation score did not improve for 6 epochs. The error definition used here is the mean absolute classification error, where the classification error, with a continuous range from − 1 to 1, represents the difference between the NN output and the target designation, i.e. whether a given patient’s total phenylephrine dosage is below (target = 0) or above (target = 1) the discrimination threshold. The absolute error range is continuous between 0 and 1, in contrast to the binary target designation, as the NN classification estimate is continuous.

**Fig. 6:**
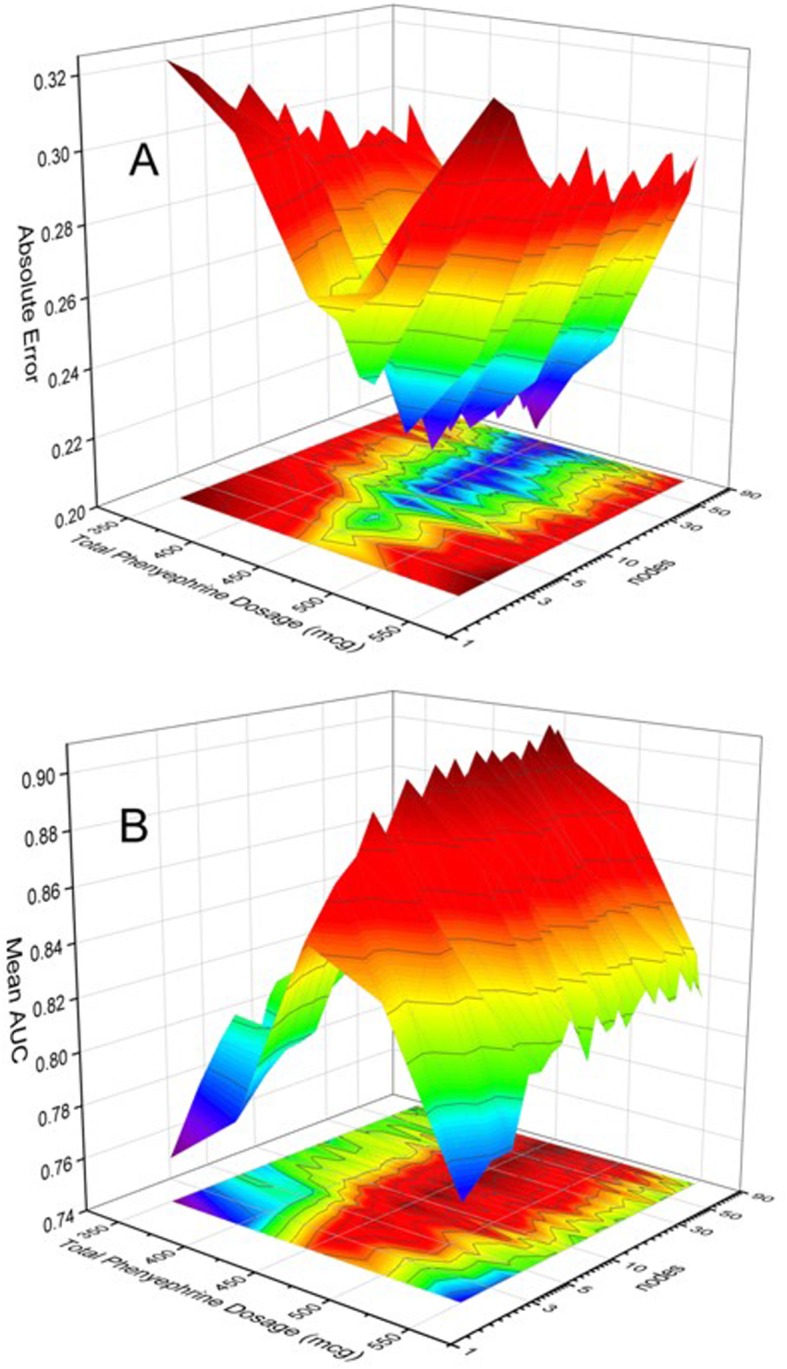
Evolution of absolute error (**a**) and mean AUC (**b**) as a function of the number of nodes of the single-layer network as well as the phenylephrine dosage. The network node axis is logarithmic to better reveal the dependence of the classification error and classification accuracy (AUC) for single-digit network nodes

In the context of assessing the categorization capability of the NN the optimum number of nodes and layers was determined, based on categorization error and mean AUC. Figure 6 presents three-dimensional graphs of the error (A) and AUC (B) evolution as a function of the number of nodes of a single hidden layer NN as well as the phenylephrine dosage. The network node axis is logarithmic to better reveal the dependence of the classification error and classification accuracy (AUC) for single-digit network nodes. The surface plots clarify that the classification error and the classification accuracy, after initially respectively decreasing/increasing with an increasing number of nodes, level off at approximately 12 nodes. This indicates that further increases in the number of nodes would only increase computational load but not enhance discrimination capability, providing the motivation for limiting the node number to 12.

**Fig. 7:**
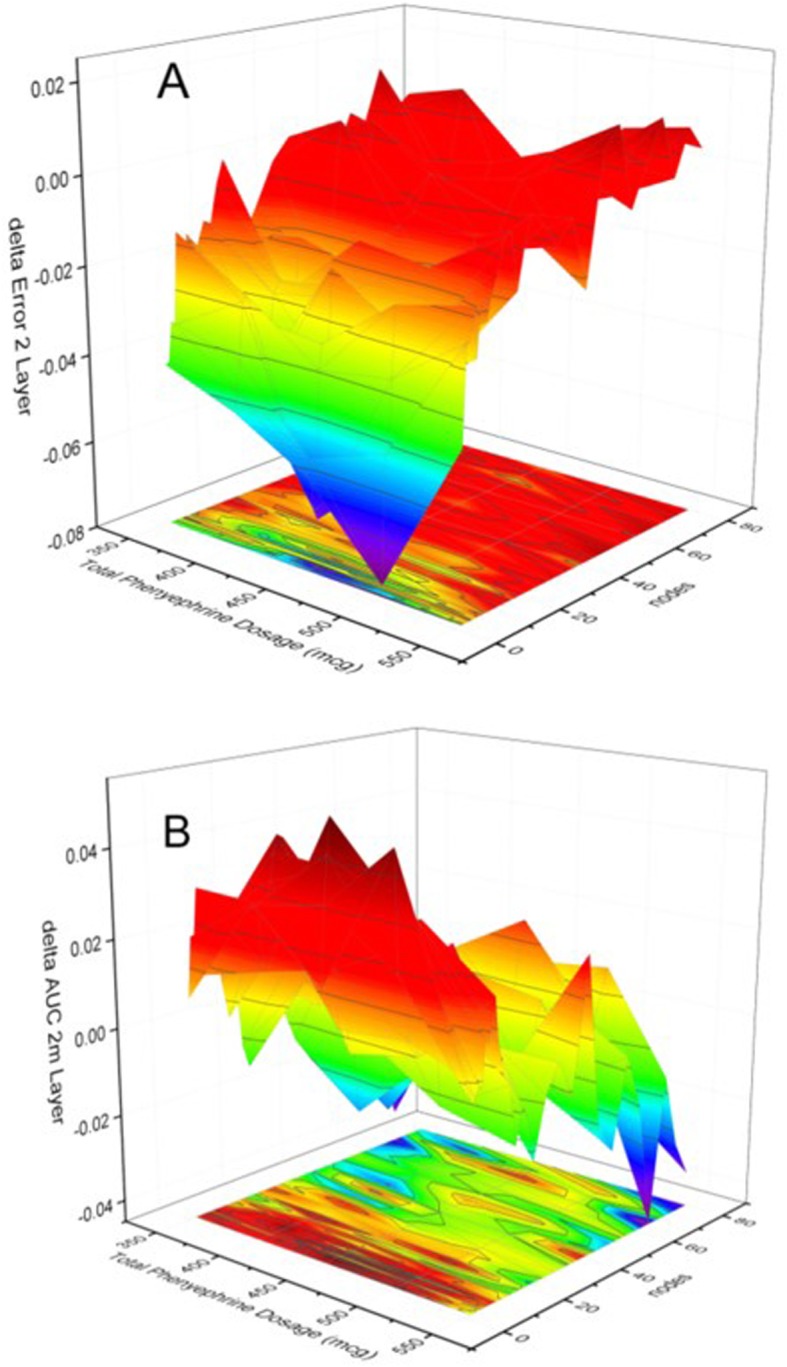
Evolution of the difference of the absolute error (**a**) and mean AUC (**b**) between the single-layer network and the two-layer network as a function of the number of nodes of the network as well as the phenylephrine dosage. Note that the error difference (A) is negative for nodes< 12, indicating that the two-layer error is larger. For nodes< 12 the AUC is smaller for the two-layer network, as indicated by the positive AUC difference. For larger node numbers there is no difference in classification performance

**Fig. 8:**
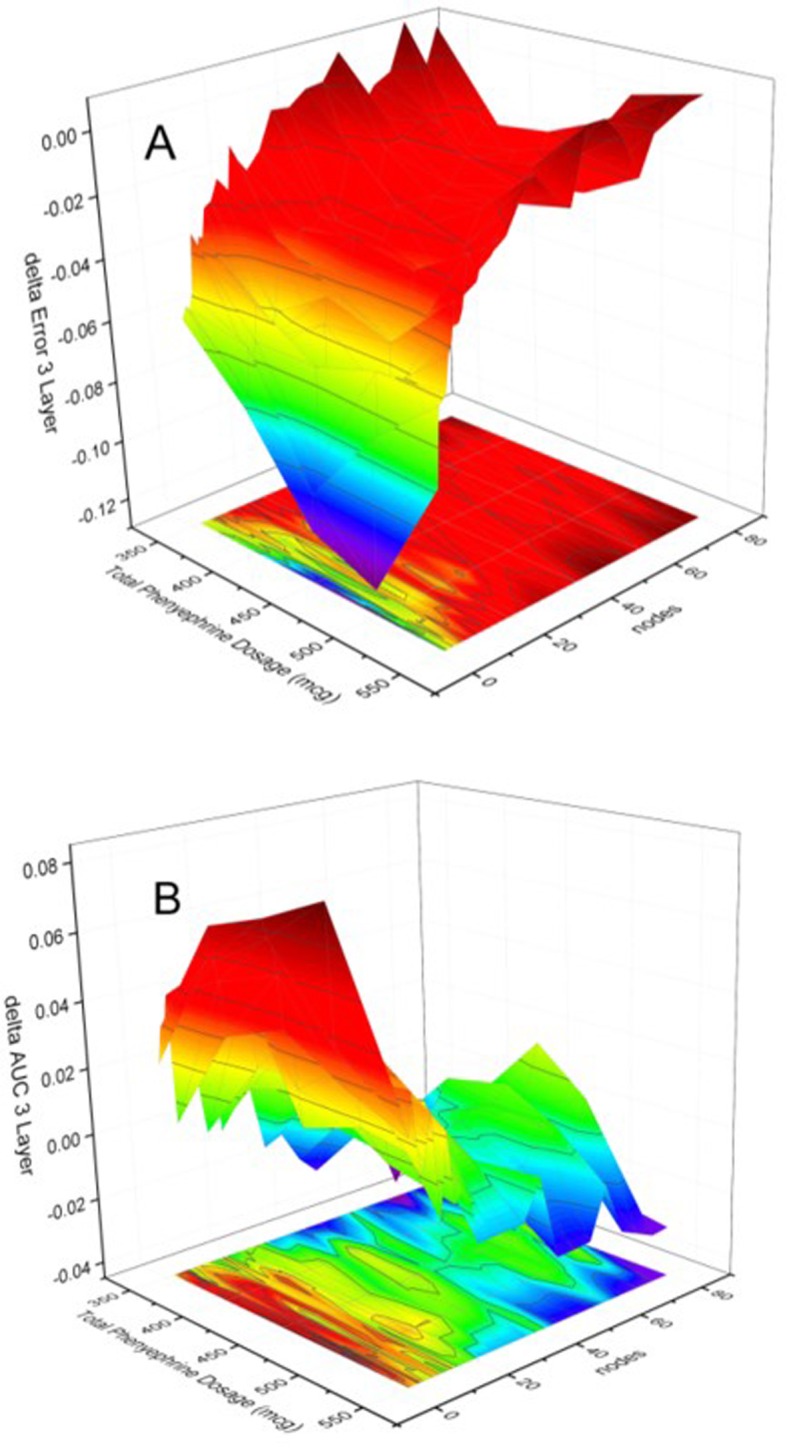
Evolution of the difference of the absolute error (**a**) and mean AUC (**b**) between the single-layer network and the three-layer network as a function of the number of nodes of the network as well as the phenylephrine dosage. Note that the error difference (A) is negative for nodes< 12, indicating that the three-layer error is larger. For nodes< 12 the AUC is smaller for the three-layer network, as indicated by the positive AUC difference. For larger node numbers there is no difference in classification performance

The results of assessing the effect of including more NN layers on categorization performance are presented in Figs. 7 & 8, which present, respectively, the *difference* between the performance of a two-layer and a three-layer 12-node NN and that of the single-layer 12-node NN shown in Fig. 6. Specifically, Figs. 7 & 8 present the *subtraction* of the categorization error (A) and of the mean AUC (B) of the respective 2-layer/3-layer NN from that of the single-layer NN. Consequently, if the performance were identical, all 4 graphs would present a plane positioned at z = 0, which is approximately the case, for the performance of both the 2-layer and the 3-layer networks, in the range of nodes > 12. For the range < 12 nodes the performance of the higher layer number networks is poorer. This is indicated by the larger errors, i.e. for both Fig. 7A and Fig. 8A the difference in error in the range < 12 nodes is negative, meaning the subtracting higher-level network error is larger than the single-layer network’s corresponding error, and by the positive AUC difference ranges displayed in Fig. 7B and Fig 8B, meaning the subtracting higher-level network AUC is smaller than that of the single-layer network, indicating the higher/better discrimination capability of the single-layer network.

The general leveling-off response characteristic for the number of nodes > 12 is observed for all phenylephrine dosage levels, however, a distinct minimum/maximum in the classification error/classification accuracy (AUC) is observed for 450 mcg, where these quantities level off at, respectively, 0.28 and 0.89 for the single-layer network. For this threshold, AUC = 0.89 (*p* < 0.001) with specificity and sensitivity, respectively, equal to 0.91 and 0.84. The resulting ROC is presented in Fig. 5, solid black curve.

An attempt was made to predict hypotension based on patient baseline information and cuff-based pre-op systolic blood pressure. The results of correlating these parameters with phenylephrine dosage administered are presented in Table 2. None achieved statistical significance, precluding any effort to build a predictive model.

**Table 2:** Correlations of patient baseline information with phenylephrine dosage

*Parameter*	*Correlation (p-value)*
Pre-op Systole	−0.17 (0.26)
Prior C/S	−0.17 (0.27)
Hypertension	−0.13 (0.39)
Diabetes Mellitus	0.05 (0.75)
Chronic obstructive pulmonary disease	No positive patients
Atrial Fibrillation	No positive patients

## Discussion

The significant findings of this study are: (1) it appears to be possible to assess the likelihood of significant post-spinal hypotension through the evaluation of coherencies in AS autocorrelation spectra and (2) this significant hemodynamic information can be obtained from non-invasive and readily obtained arterial pressure pulse information.

The results presented here add to the previous research in that multiple studies have identified changes in AS in comparisons of pregnant versus normal women as well as over the course of pregnancy. A prior study by Osman determined that arterial stiffness changes sinusoidally during pregnancy with an overall mean pulse wave velocity (PWV), the Gold Standard surrogate parameter, of 7.81 m/s significantly lower than the measured 10.0 m/s in non-pregnant women [13]. Different mechanisms have been proposed to explain the changes in PWV throughout pregnancy. While the initial drop may be due to changes of vaso-active substances such as nitric oxide (NO) [23, 24], progesterone, relaxin, the changes may also be related to volume expansion [25], while the inhibition of NO, an increase in cardiac output or increased circulatory volume [26] could be responsible for the increase that is observed from mid-trimester to term [27].

A question in the context of these findings, which were obtained during normal gestational evolution, is whether these changes in AS, and probably other hemodynamic parameters, indicate that certain pregnant women are more prone to developing hemodynamic instabilities in response to a significant stressor, such as spinal anesthesia? Our results suggest that increased coherence in AS may be such a marker. If increased coherence is interpreted as decreased spontaneous variability, the concept of a predictive model of this specific case of hypotension becomes more plausible. Variabilities in different physiological parameters, heart rate variability for example, have been shown to decrease prior to hemodynamic crises, such as a hypotensive event, as compensatory mechanisms in the cardiovascular system try to maintain stability [28].

Comparing the classification performance of a discrete parameterization of a physiological data set such as the autocorrelation spectra used here with that of a generalized pattern recognition approach such as NNs is useful because the generalized approach can provide an assessment of the totality of available features that would aid successful classification. This is likely why the NN approach was more successful at classification than the discrete approach, although the small degree of improvement is also indicative, as is discussed below. The NN utilized a set of distinguishing features in its optimization of classification, as opposed to a single measure. As an example, we investigated utilizing the first crossing of the autocorrelation spectrum as a basis of classification. However, as a single-variable classification attempt the approach yielded only an AUC = 0.6. Other features to add to the discrete model could include, for example, distinct frequency components in autocorrelation spectrum. The NN likely identified a combination of these multiple features and others.

However, given that the discrete feature classification approach yielded comparable performance results with those of the NN suggests that the feature that the absolute value integration was designed to quantify, primarily the amplitude of the AS modulations, represents a significant portion of the classification potential available in the AS autocorrelation spectra. In fact, and this point was examined as part of the validation analysis for the number of nodes and layers used in the NN, the lack of significant improvement in the classification performance of the NN over that of the discrete feature suggests a dearth of additional available features overall. Specifically, increasing the number of nodes past the threshold value of 12 did not improve performance, i.e. both classification error and accuracy remained flat and significantly different from what would indicate perfect classification, addressing over-fitting concerns.

The same consideration applies to the results of expanding the NN to include multiple layers, which potentially provides access to more refined categorization opportunities in the combinations of inputs. The fact that these network expansions yielded no improvement in categorization capability again suggests that hidden features that could be used to improve performance are not present in the input AS autocorrelation spectra.

These considerations in turn suggest that the addition of complementary data sources, such as for example heart rate variability spectra, will be required to significantly enhance the classification capability of either the NN or the discrete approach.

The results presented here suggest that the NN approach, at least at the implementation level of a clinically relevant prediction algorithm, is somewhat superior to the discrete feature approach, providing implicit access to a plurality of features and, presumably, combinations thereof. In addition, the expansion of the approach to include the submission of other physiological data signals to the network can be readily envisioned and has been implemented in the context of general hypotension prediction by others [7]. In the context of gaining understanding of the underlying physiological mechanisms, however, discrete feature identification will continue to be relevant for reasons we present below.

The results presented here fit into the larger picture of the evolving and increasingly successful attempt to predict impending hypotension in critical clinical settings from arterial waveforms [6, 7]. However, these recent studies have focused on using the discrimination capability of NNs primarily because traditionally hemodynamic parameters such as heart rate variability [29, 30], stroke volume variability [31], arterial stiffness [32] or pulsatility indices [33] have been usually obtained as static single measurements that do not lend themselves to continuous, discrete monitoring. Nonetheless, it is very plausible that the NNs utilize the listed hemodynamic parameters in their categorization assessment through their effect on the arterial pulse, but the dependence is hidden. Problems arise when the NN approach fails in the absence of a more concrete underlying physiological model, because trouble-shooting a NN is not an option, only to include more data and hope that a re-learned NN will perform better. Our work suggests that there may be a useful intersection between discrete assessments, particularly as the extraction of traditional hemodynamic parameters as beat-by-beat pulse features matures, and the general and considerable capabilities of the NN.

From the clinical point of view the incremental value of the proposed approach, be it based on discrete feature- or NN-based discrimination, is promising. Knowledge of pre-op blood pressures, specific patient conditions and/or comorbidities, which in general may or may not be completely available, in the cohort studied had no predictive value regarding post-induction hypotension, the blood pressure results specifically being in line with the results of others [6]. On the one hand a number of potential separate tests could be performed pre-op to assess certain hemodynamic parameters, but this approach is limited if even feasible. On the other hand is the proposed approach, with its simplicity particularly in the context of clinical workflow, since the arterial pulse information is collected continuously from the finger cuff-based sensing system, providing a data stream of high hemodynamic information content, irrespective of availability of patient history.

The focus of our preliminary study was to develop a predictive model of vasopressor requirements in this specific obstetric population. The ability to predict with advanced warning adverse events (hypotension) in this vulnerable population is an important step in patient care. If specific pre-operative interventions (e.g. additional intravenous fluid) in patients predicted to need substantial vasopressor administration can minimize the need for pharmacologic intervention, it would be of significant clinical value. A recent review article has shown that vasopressor interventions can adversely affect the fetal acid base status [34].

The population of pregnant patients undergoing C/S was chosen primarily because of the high frequency of adverse events requiring vasopressor support in this vulnerable population (92%). This allowed us to predict specific dosing thresholds of phenylephrine required In future work, we also want to assess the ability of our approach to predict hypotension in other surgical procedures where hypotension is a rarer event.

In the context of different clinical applications the sensitivity versus specificity aspects of the presented analyses should be considered. While the NN-based analysis demonstrated slightly better overall discrimination capability, closer examination of the corner regions of Fig. 5 suggests that the discrete feature approach may provide advantages were specificity is more critical. Increased perfection of a discrimination test is characterized in the ROC curve by an increasingly steep rise as a function of the false positive rate (1-specificity), and it is in that region of the ROC curves shown in Fig. 5 that the discrete feature approach appears to outperform the NN discrimination. In clinical applications where hypotension is a rarer event, the evolution of that aspect of the two discrimination analysis approaches will be or great interest as more data becomes available.

Further refinements and improvements to the predictive approach are possible as the CT system also provides access to heart rate variability, respiration signals and other hemodynamic parameters, such as cardiac output.

The size of the patient population and the classification performance of the parallel approaches support the statistical significance of the presented results, suggesting that it is unlikely that the analysis was optimized for this set of patient data, discounting the zero-effect hypothesis. Further studies will investigate the performance of the approach with scaling and additional physiological variables.

## Conclusions

This pilot study has demonstrated that increased coherence in AS variability obtained from the pulse wave analysis of a continuous non-invasive blood pressure device appears to be an effective predictor of hypotension after spinal anesthesia in the obstetrics population undergoing C/S. Autocorrelation spectra were found to be valid biomarkers for this analysis, and it was possible to correlate spectra coherence with specific clinical dosing outcomes. These are significant findings since post-spinal hypotension is a common clinical scenario that has the potential to affect the mother and baby. Next steps are to examine if specific interventions can be used preoperatively to minimize the need for intra-operative vasopressors. We also want to assess the ability of our approach to predict hypotension in other surgical procedures where hypotension is a rarer event.

## Data Availability

Data will be made available upon request by contacting the corresponding author Irwin Gratz, DO (T: + 1 856–968-8527, E: gratz-irwin@cooperhealth.edu).

## Abbreviations

C/S: Cesarean section
AS: Arterial stiffness
NIBP: Noninvasive blood pressure
MAP: Mean arterial pressure
CT: CareTaker
PDA: Pulse decomposition analysis
P1: Left ventricular ejection pulse
P2: Renal reflection pulse
P3: Iliac reflection pulse
ROC: Receiver operating characteristic
AUC: Area under curve
PMV: Mean pulse velocity
PWV: Pulse wave velocity
NO: Nitric oxide
NN: Neural network
